# Gestational weight gain and foetal acidosis in vaginal and caesarean deliveries: The Japan Environment and Children’s Study

**DOI:** 10.1038/s41598-020-77429-9

**Published:** 2020-11-23

**Authors:** Tsuyoshi Murata, Hyo Kyozuka, Akiko Yamaguchi, Toma Fukuda, Shun Yasuda, Akiko Sato, Yuka Ogata, Kosei Shinoki, Mitsuaki Hosoya, Seiji Yasumura, Koichi Hashimoto, Hidekazu Nishigori, Keiya Fujimori, The Japan Environment, Children’s Study (JECS) Group, Michihiro Kamijima, Shin Yamazaki, Yukihiro Ohya, Reiko Kishi, Nobuo Yaegashi, Chisato Mori, Shuichi Ito, Zentaro Yamagata, Hidekuni Inadera, Takeo Nakayama, Hiroyasu Iso, Masayuki Shima, Youichi Kurozawa, Narufumi Suganuma, Koichi Kusuhara, Takahiko Katoh

**Affiliations:** 1 Fukushima Regional Center for the Japan Environmental and Children’s Study, Fukushima Medical University, 1 Hikarigaoka, Fukushima, 960-1295 Japan; 2 Department of Obstetrics and Gynecology, School of Medicine, Fukushima Medical University, 1 Hikarigaoka, Fukushima, 960-1295 Japan; 3 Department of Pediatrics, Fukushima Medical University School of Medicine, 1 Hikarigaoka, Fukushima, 960-1295 Japan; 4 Department of Public Health, Fukushima Medical University School of Medicine, 1 Hikarigaoka, Fukushima, 960-1295 Japan; 5 Fukushima Medical Center for Children and Women, Fukushima Medical University, 1 Hikarigaoka, Fukushima, 960-1295 Japan; 6 Graduate School of Medical Sciences Department of Occupational and Environmental Health, Nagoya City University, 1 Kawasumi, Mizuho-cho, Mizuho-ku, Nagoya, Aichi 467-8601 Japan; 7 National Institute for Environmental Studies, Tsukuba, Japan; 8 National Center for Child Health and Development, Tokyo, Japan; 9 Hokkaido University, Sapporo, Japan; 10 Tohoku University, Sendai, Japan; 11 Chiba University, Chiba, Japan; 12 Yokohama City University, Yokohama, Japan; 13 University of Yamanashi, Chuo, Japan; 14 University of Toyama, Toyama, Japan; 15 Kyoto University, Kyoto, Japan; 16 Osaka University, Suita, Japan; 17 Hyogo College of Medicine, Nishinomiya, Japan; 18 Tottori University, Yonago, Japan; 19 Kochi University, Nankoku, Japan; 20 University of Occupational and Environmental Health, Kitakyushu, Japan; 21 Kumamoto University, Kumamoto, Japan

**Keywords:** Disease prevention, Patient education, Weight management, Epidemiology, Risk factors

## Abstract

Inappropriate gestational weight gain (GWG), either above or below the recommended values, has been associated with an increased risk of adverse obstetric outcomes. To evaluate the risks of GWG for foetal acidosis according to pre-pregnancy body mass index (BMI) and mode of delivery, we analysed women with singleton pregnancies between 2011 and 2014 in the Japan Environment and Children’s Study. Participants (n = 71,799) were categorised according to pre-pregnancy BMI. GWG was categorised into insufficient, appropriate, or excessive. Foetal acidosis was defined as umbilical artery pH (UmA-pH) < 7.20 or < 7.10. Multiple logistic regressions were performed for each BMI category to identify the risks of GWG for foetal acidosis, accounting for the mode of delivery. Excessive GWG was significantly associated with increased foetal acidosis in overweight women and in women whose pre-pregnancy BMI was 23.0–25.0 kg/m^2^ especially in those with vaginal deliveries. Conversely, excessive GWG was not significantly associated with increased foetal acidosis in obese women and in women whose pre-pregnancy BMI was  ≥ 25.0 kg/m^2^.

## Introduction

Inappropriate gestational weight gain (GWG), which is greater or lower than the recommended values, has been associated with an increased risk of adverse obstetric outcomes, such as preterm births (PTB), small-for-gestational age (SGA) infants, and macrosomia^1^. The recommendation made by the Institute of Medicine (IOM; now known as the National Academy of Medicine) was a GWG of 12.5–18 kg for underweight women (pre-pregnancy body mass index (BMI) < 18.5 kg/m^2^), 11.5–16 kg for normal-weight women (BMI 18.5–24.9 kg/m^2^), 7–11 kg for overweight women (BMI 25.0–29.9 kg/m^2^), and 5–9 kg for obese women (BMI ≥ 30.0 kg/m^2^)^2–4^. A previous study has reported the relationship between GWG and an increased risk of severe birth-asphyxia-related outcomes, such as low Apgar score, meconium aspiration, and neonatal seizures^5^. Foetal asphyxia could lead to neuronal injury, long-term morbidity, and death^6–8^.

Foetal acidosis is a major factor associated with birth asphyxia, generally resulting from the interruption of placental blood flow and subsequent foetal hypoxia and hypercarbia^9–11^. A low umbilical artery pH (UmA-pH) is significantly associated with neonatal mortality^10^ and metabolic acidosis is significantly associated with foetal hypoxic-ischaemic brain injury^12^. Thus, reducing foetal acidosis during antepartum and intrapartum is an important issue to resolve^13^.

Although previous studies have shown an association between GWG and adverse perinatal outcomes, including foetal acidosis^1,5,14–17^, there are no reports indicating an association between GWG and foetal acidosis based on the GWG validated for each pre-pregnancy BMI category. Additionally, the effects of the mode of delivery on foetal acidosis have not been clarified in this setting. As appropriate gestational bodyweight control is desired for achieving favourable pregnancy outcomes^18^, obtaining data regarding GWG based on each pre-pregnancy BMI category and foetal acidosis, with consideration of the mode of delivery, is of great concern to perinatal physicians.

We aimed to examine GWG as a risk factor of foetal acidosis, according to established pre-pregnancy BMI categories and the mode of delivery, using a large Japanese cohort study: the Japan Environment and Children’s Study (JECS).

## Results

**Figure 1:**
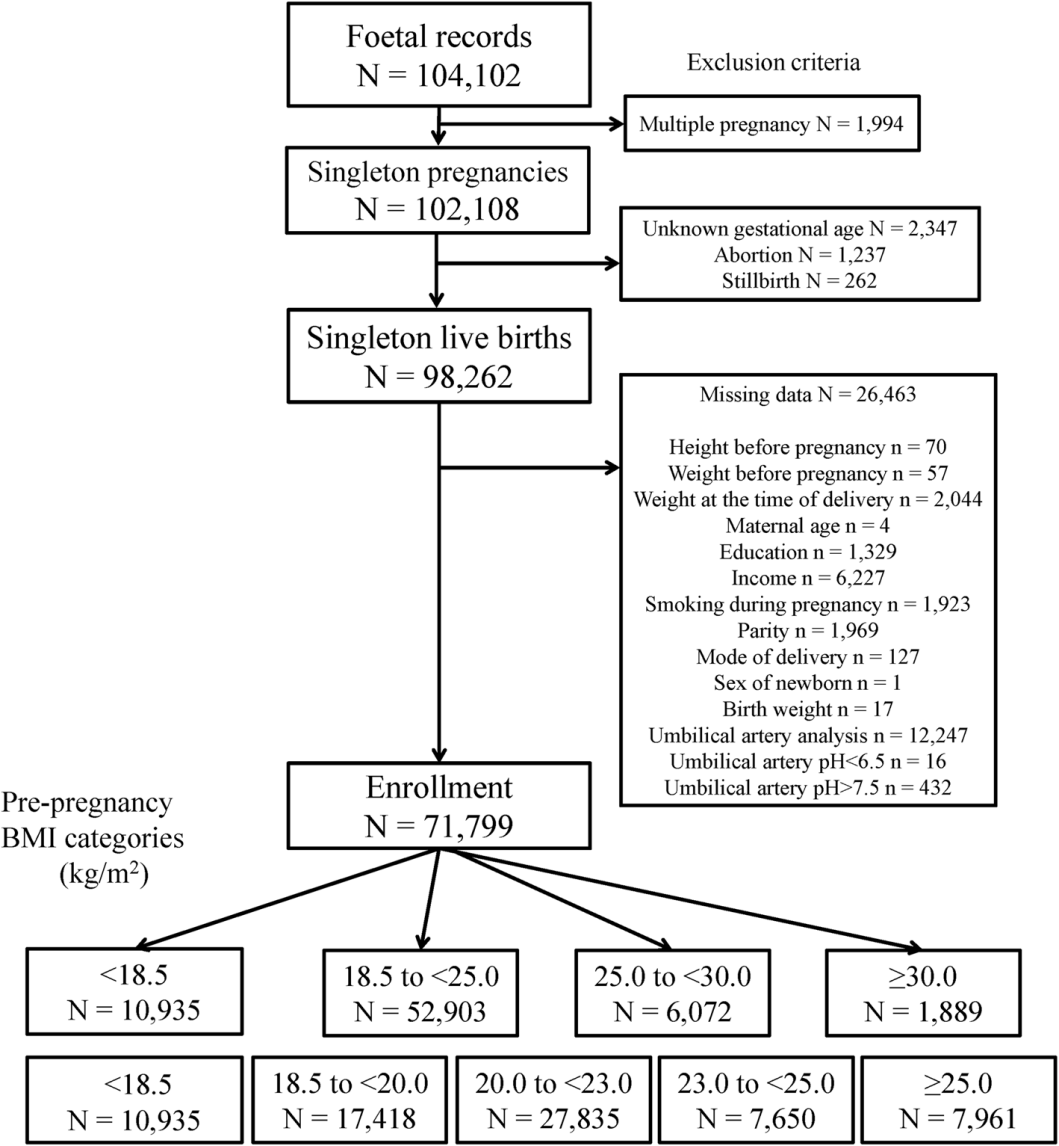
Flowchart of study enrolment.

Among the 104,102 foetal records from 2011 to 2014 in the JECS, 71,799 were eligible for the analysis (Fig. 1). There were 10,935 (15.2%), 52,903 (73.7%), 6072 (8.5%), and 1889 (2.6%) births in IOM-criteria-based BMI groups A (GA, < 18.5 kg/m^2^), B (GB, 18.5–< 25.0 kg/m^2^), C (GC, 25.0–< 30.0 kg/m^2^; overweight), and D (GD ≥ 30.0 kg/m^2^; obese), respectively; and 10,935 (15.2%), 17,418 (24.3%), 27,835 (38.8%), 7650 (10.7%), and 7961 (11.1%) births in Japanese-criterial-based BMI groups 1 (G1, < 18.5 kg/m^2^), 2 (G2, 18.5–< 20.0 kg/m^2^), 3 (G3, 20.0–< 23.0 kg/m^2^), 4 (G4, 23.0–< 25.0 kg/m^2^), and 5 (G5, ≥ 25.0 kg/m^2^), respectively. There were 58,345 (81.3%) and 13,454 (18.7%) vaginal and caesarean deliveries, respectively. In GD and G5, the ratio of PTB, caesarean delivery, UmA-pH < 7.20, and average birth weight were the highest; conversely, GWG was the lowest (Table 1).

**Table 1:** Maternal background according to pre-pregnancy body mass index based on 2009 IOM guidelines and Japanese criteria^28^.

Group	GA	GB	GC	GD	P value	
BMI category (kg/m^2^)	< 18.5	18.5–< 25.0	25.0–< 30.0	≥ 30.0		
No. patients	10,935 (15.2%)	52,903 (73.7%)	6072 (8.5%)	1889 (2.6%)		
**Maternal background**						
Maternal age (years), %						
< 20	0.9	0.5	0.5	0.3	< 0.001	
20–29	41.9	34.9	32.0	31.6		
> 30	57.2	64.5	67.6	68.2		
Maternal education (years), %						
< 10	5.1	4.0	6.0	8.9	< 0.001	
10–12	30.9	29.9	38.0	43.5		
13–16	62.5	64.6	54.9	47.0		
> 17	1.5	1.4	1.1	0.5		
Annual household income (JPY), %						
< 2,000,000	6.0	5.2	7.6	10.3	< 0.001	
2,000,000–5,999,999	67.3	67.1	70.4	72.6		
6,000,000–9,999,999	22.1	23.3	18.6	15.0		
> 10,000,000	4.6	4.4	3.4	2.1		
Smoking during pregnancy, %	5.0	4.2	6.5	7.8	< 0.001	
Primiparous, %	43.4	40.1	34.4	34.0	< 0.001	
**Obstetrics outcomes**						
Gestational age, week (mean ± SD)	38.7 (1.5)	38.9 (1.5)	38.8 (1.6)	38.6 (1.9)	< 0.001	
PTB before 37 weeks, %	5.0	4.0	5.5	7.3	< 0.001	
Birth weight, g (mean ± SD)	2925 (388)	3038 (401)	3115 (454)	3157 (508)	< 0.001	
SGA infants, %	7.3	4.6	4.0	3.3	< 0.001	
Gestational weight gain, kg (mean ± SD)	10.8 (3.5)	10.5 (3.8)	8.6 (10.0)	5.2 (5.7)	< 0.001	
Caesarean delivery, % (n)	14.8 (1614)	18.0 (9506)	27.3 (1657)	35.8 (677)	< 0.001	
UmA-pH < 7.2, %	6.2	6.2	6.9	7.5	0.037	
UmA-pH < 7.1, %	1.1	1.2	1.3	1.3	0.554	

Group	G1	G2	G3	G4	G5	P value
BMI category (kg/m^2^)	< 18.5	18.5–< 20.0	20–< 23.0	23.0–< 25.0	≥ 25	
No. patients	10,935 (15.2%)	17,418 (24.3%)	27,835 (38.8%)	7650 (10.7%)	7961 (11.1%)	
**Maternal background**						
Maternal age (years), %						
< 20	0.9	0.7	0.5	0.3	0.4	< 0.001
20–29	41.9	36.8	34.6	31.9	31.9	
> 30	57.2	62.6	64.9	67.8	67.7	
Maternal education (years), %						
< 10	5.1	3.8	3.9	4.4	6.7	< 0.001
10–12	30.9	28.5	29.8	33.7	39.3	
13–16	62.5	66.1	64.8	60.8	53.0	
> 17	1.5	1.6	1.4	1.2	1.0	
Annual household income, %						
< 2,000,000	6.0	5.0	5.1	6.0	8.2	< 0.001
2,000,000–5,999,999	67.3	65.6	67.3	69.7	70.9	
6,000,000–9,999,999	22.1	24.3	23.3	21.2	17.8	
> 10,000,000	4.6	5.0	4.4	3.0	3.1	
Smoking during pregnancy, %	5.0	3.9	4.1	4.9	6.8	< 0.001
Primiparous, %	43.4	42.6	39.4	36.6	34.3	< 0.001
**Obstetrics outcomes**						
Gestational age, week (mean ± SD)	38.7 (1.5)	38.9 (1.4)	38.9 (1.5)	38.9 (1.5)	38.8 (1.7)	< 0.001
PTB before 37 weeks, %	5.0	4.0	4.0	4.2	5.9	< 0.001
Birth weight, g (mean ± SD)	2925 (388)	2997 (387)	3049 (401)	3095 (421)	3125 (467)	< 0.001
SGA infants, %	7.3	5.4	4.4	3.8	3.8	< 0.001
Gestational weight gain, kg (mean ± SD)	10.8 (3.5)	10.7 (3.5)	10.6 (3.8)	10.0 (4.4)	7.8 (9.3)	< 0.001
Caesarean delivery, % (n)	14.8 (1614)	15.3 (2666)	18.4 (5132)	22.3 (1708)	29.3 (2334)	< 0.001
UmA-pH < 7.2, %	6.2	5.9	6.4	6.4	7.0	0.014
UmA-pH < 7.1, %	1.1	1.1	1.2	1.2	1.3	0.355

One-way analysis of variance, Kruskal-Wallis test and the Chi-square test were used to compare continuous and categorical variables, respectively. *BMI* body mass index, *G1* group 1, *G2* group 2, *G3* group 3, *G4* group 4, *G5* group 5, *GA* group A, *GB* group B, *GC* group C, *GD* group D, *IOM* Institute of Medicine, *JPY* Japanese yen, *PTB* preterm birth, *SGA* small-for-gestational age, *UmA-pH* umbilical artery pH.

The adjusted odds ratio (aOR) of UmA-pH < 7.10 of patients with excessive GWG in GC were 2.07 (1.19–3.60) in Model 1 and 2.08 (95% confidence interval [CI], 1.19–3.61) in Models 2 and 3. The aORs of UmA-pH < 7.20 of patients with excessive GWG in G4 were 1.25 (95% CI 1.01–1.54) in Model 2 and 1.25 (95% CI 1.02–1.54) in Model 3. The aORs of UmA-pH < 7.10 of patients with excessive GWG in G4 were 1.66 (95% CI 1.01–2.74), 1.72 (95% CI 1.04–2.84), and 1.71 (95% CI 1.04–2.83) in Models 1, 2, and 3, respectively. Conversely, the aORs of UmA-pH < 7.20 of patients with insufficient GWG in G1 were 0.83 (95% CI 0.69–0.99) in Models 2 and 3 (Tables 2, 3).

**Table 2:** Adjusted odds ratios^a^ and 95% confidence intervals for foetal acidosis based on gestational weight gain described in the 2009 IOM guidelines.

BMI category (kg/m^2^)	Gestational weight gain		
	Insufficient	Appropriate	Excessive
**UmA-pH < 7.2**			
Group A; BMI < 18.5 (n = 10,935)	n = 7920	n = 2759	n = 256
Model 1 aOR (95% CI)	0.92 (0.77–1.10)	Ref	1.13 (0.69–1.83)
Model 2 aOR (95% CI)	0.88 (0.73–1.05)	Ref	1.14 (0.70–1.85)
Model 3 aOR (95% CI)	0.88 (0.73–1.05)	Ref	1.15 (0.71–1.87)
Group B; BMI 18.5–< 25.0 (n = 52,903)	n = 31,671	n = 17,489	n = 3743
Model 1 aOR (95% CI)	0.97 (0.90–1.05)	Ref	1.10 (0.96–1.26)
Model 2 aOR (95% CI)	0.96 (0.89–1.04)	Ref	1.11 (0.96–1.27)
Model 3 aOR (95% CI)	0.96 (0.89–1.04)	Ref	1.11 (0.97–1.28)
Group C; BMI 25.0–< 30.0 (n = 6072)	n = 2125	n = 2383	n = 1564
Model 1 aOR (95% CI)	0.94 (0.73–1.19)	Ref	1.27 (0.99–1.62)
Model 2 aOR (95% CI)	0.92 (0.72–1.18)	Ref	1.27 (0.99–1.62)
Model 3 aOR (95% CI)	0.92 (0.72–1.18)	Ref	1.27 (0.99–1.62)
Group D; BMI ≥ 30 (n = 1889)	n = 888	n = 567	n = 434
Model 1 aOR (95% CI)	0.97 (0.65–1.46)	Ref	0.98 (0.61–1.57)
Model 2 aOR (95% CI)	0.96 (0.64–1.43)	Ref	0.99 (0.62–1.60)
Model 3 aOR (95% CI)	0.96 (0.64–1.43)	Ref	0.99 (0.62–1.60)
**UmA-pH < 7.1**			
Group A; BMI < 18.5 (n = 10,935)	n = 7920	n = 2759	n = 256
Model 1 aOR (95% CI)	0.93 (0.61–1.42)	Ref	1.71 (0.66–4.46)
Model 2 aOR (95% CI)	0.81 (0.53–1.25)	Ref	1.75 (0.67–4.58)
Model 3 aOR (95% CI)	0.81 (0.53–1.25)	Ref	1.71 (0.66–4.48)
Group B; BMI 18.5–< 25.0 (n = 52,903)	n = 31,671	n = 17,489	n = 3743
Model 1 aOR (95% CI)	1.11 (0.93–1.33)	Ref	1.33 (0.98–1.80)
Model 2 aOR (95% CI)	1.07 (0.89–1.28)	Ref	1.35 (0.99–1.83)
Model 3 aOR (95% CI)	1.07 (0.89–1.28)	Ref	1.35 (0.99–1.82)
Group C; BMI 25.0–< 30.0 (n = 6072)	n = 2125	n = 2383	n = 1564
Model 1 aOR (95% CI)	1.33 (0.75–2.38)	Ref	2.07 (1.19–3.60)
Model 2 aOR (95% CI)	1.26 (0.70–2.25)	Ref	2.08 (1.19–3.61)
Model 3 aOR (95% CI)	1.26 (0.70–2.25)	Ref	2.08 (1.19–3.61)
Group D; BMI ≥ 30 (n = 1889)	n = 888	n = 567	n = 434
Model 1 aOR (95% CI)	1.94 (0.62–6.08)	Ref	2.64 (0.80–8.71)
Model 2 aOR (95% CI)	1.80 (0.57–5.67)	Ref	2.71 (0.82–9.00)
Model 3 aOR (95% CI)	1.80 (0.57–5.67)	Ref	2.71 (0.82–9.00)

Logistic regression models were used to calculate the adjusted odds ratios and 95% confidence intervals for UmA-pH < 7.20, with women with appropriate gestational weight gain as the reference. *BMI* body mass index, *IOM* Institute of Medicine, *UmA-pH* umbilical artery pH.

^a^Model 1 adjusted for maternal age, maternal education, annual household income, maternal smoking during pregnancy, and parity. Model 2 adjusted for covariates in Model 1 and preterm birth and small-for-gestational age infants. Model 3 adjusted for covariates in Model 2 and mode of delivery.

**Table 3:** Adjusted odds ratios^a^ and 95% confidence intervals for foetal acidosis based on gestational weight gain described in Japanese criteria^28^.

BMI category (kg/m^2^)	Gestational weight gain		
	Insufficient	Appropriate	Excessive
**UmA-pH < 7.2**			
Group 1; BMI < 18.5 (n = 10,935)	n = 5550	n = 3316	n = 2069
Model 1 aOR (95% CI)	0.88 (0.73–1.05)	Ref	0.98 (0.79–1.23)
Model 2 aOR (95% CI)	0.83 (0.69–0.99)	Ref	1.00 (0.80–1.25)
Model 3 aOR (95% CI)	0.83 (0.69–0.99)	Ref	1.00 (0.80–1.25)
Group 2; BMI 18.5–< 20.0 (n = 17,418)	n = 6310	n = 6229	n = 4879
Model 1 aOR (95% CI)	0.99 (0.85–1.15)	Ref	1.10 (0.94–1.29)
Model 2 aOR (95% CI)	0.98 (0.84–1.14)	Ref	1.10 (0.94–1.30)
Model 3 aOR (95% CI)	0.98 (0.84–1.14)	Ref	1.11 (0.95–1.30)
Group 3; BMI 20.0–< 23.0 (n = 27,835)	n = 7248	n = 9849	n = 10,738
Model 1 aOR (95% CI)	0.95 (0.84–1.08)	Ref	1.01 (0.90–1.13)
Model 2 aOR (95% CI)	0.94 (0.83–1.07)	Ref	1.01 (0.91–1.13)
Model 3 aOR (95% CI)	0.94 (0.83–1.07)	Ref	1.02 (0.91–1.14)
Group 4; BMI 23.0–< 25.0 (n = 7650)	n = 1054	n = 2622	n = 3974
Model 1 aOR (95% CI)	0.94 (0.69–1.29)	Ref	1.21 (0.99–1.49)
Model 2 aOR (95% CI)	0.91 (0.66–1.24)	Ref	1.25 (1.01–1.54)
Model 3 aOR (95% CI)	0.91 (0.66–1.25)	Ref	1.25 (1.02–1.54)
Group 5; BMI ≥ 25.0 (n = 7961)	n = 893	n = 2488	n = 4580
Model 1 aOR (95% CI)	0.87 (0.64–1.20)	Ref	1.05 (0.86–1.27)
Model 2 aOR (95% CI)	0.86 (0.63–1.18)	Ref	1.06 (0.88–1.29)
Model 3 aOR (95% CI)	0.87 (0.63–1.19)	Ref	1.07 (0.88–1.29)
**UmA-pH < 7.1**			
Group 1; BMI < 18.5 (n = 10,935)	n = 5550	n = 3316	n = 2069
Model 1 aOR (95% CI)	1.23 (0.79–1.91)	Ref	1.41 (0.83–2.39)
Model 2 aOR (95% CI)	1.07 (0.68–1.67)	Ref	1.48 (0.87–2.50)
Model 3 aOR (95% CI)	1.07 (0.68–1.67)	Ref	1.47 (0.87–2.49)
Group 2; BMI 18.5–< 20.0 (n = 17,418)	n = 6310	n = 6229	n = 4879
Model 1 aOR (95% CI)	1.00 (0.71–1.43)	Ref	1.20 (0.84–1.72)
Model 2 aOR (95% CI)	0.95 (0.66–1.35)	Ref	1.24 (0.87–1.78)
Model 3 aOR (95% CI)	0.95 (0.66–1.35)	Ref	1.24 (0.86–1.78)
Group 3; BMI 20.0–< 23.0 (n = 27,835)	n = 7248	n = 9849	n = 10,738
Model 1 aOR (95% CI)	0.94 (0.71–1.25)	Ref	1.03 (0.81–1.32)
Model 2 aOR (95% CI)	0.89 (0.67–1.19)	Ref	1.06 (0.82–1.35)
Model 3 aOR (95% CI)	0.89 (0.67–1.19)	Ref	1.05 (0.82–1.35)
Group 4; BMI 23.0–< 25.0 (n = 7650)	n = 1054	n = 2622	n = 3974
Model 1 aOR (95% CI)	1.56 (0.79–3.05)	Ref	1.66 (1.01–2.74)
Model 2 aOR (95% CI)	1.50 (0.76–2.96)	Ref	1.72 (1.04–2.84)
Model 3 aOR (95% CI)	1.50 (0.76–2.96)	Ref	1.71 (1.04–2.83)
Group 5; BMI ≥ 25 (n = 7961)	n = 893	n = 2488	n = 4,580
Model 1 aOR (95% CI)	0.57 (0.25–1.30)	Ref	1.00 (0.66–1.53)
Model 2 aOR (95% CI)	0.54 (0.24–1.24)	Ref	1.07 (0.70–1.63)
Model 3 aOR (95% CI)	0.54 (0.24–1.24)	Ref	1.07 (0.70–1.63)

Logistic regression models were used to calculate the adjusted odds ratios and 95% confidence intervals for UmA-pH < 7.20 and for UmA-pH < 7.10, with women with appropriate gestational weight gain as the reference. *BMI* body mass index, *UmA-pH* umbilical artery pH.

^a^Model 1 adjusted for maternal age, maternal education, annual household income, maternal smoking during pregnancy, and parity. Model 2 adjusted for covariates in Model 1 and preterm birth and small-for-gestational age infants. Model 3 adjusted for covariates in Model 2 and mode of delivery.

The aORs of UmA-pH < 7.20 and UmA-pH < 7.10 of patients with excessive GWG in G4 with vaginal deliveries were 1.31 (95% CI 1.04–1.62) and 2.08 (95% CI 1.13–3.83), respectively. Conversely, the aOR of UmA-pH < 7.10 of patients with insufficient GWG in G5 with vaginal deliveries was 0.29 (95% CI 0.09–0.97) (Table 4). There was no association between GWG and the incidence of foetal acidosis in participants who underwent caesarean section (Table 5).

**Table 4:** Adjusted odds ratios^a^ and 95% confidence intervals for foetal acidosis based on gestational weight gain with vaginal deliveries according to Japanese criteria^28^.

BMI category (kg/m^2^)	Gestational weight gain		
	Insufficient	Appropriate	Excessive
**UmA-pH < 7.2**			
Group 1; BMI < 18.5 (n = 9321)	n = 4664	n = 2871	n = 1786
aOR (95% CI)	0.85 (0.70–1.04)	Ref	1.03 (0.81–1.30)
Group 2; BMI 18.5–< 20.0 (n = 14,752)	n = 5261	n = 5294	n = 4197
aOR (95% CI)	0.97 (0.83–1.15)	Ref	1.12 (0.94–1.32)
Group 3; BMI 20.0–< 23.0 (n = 22,703)	n = 5807	n = 8099	n = 8797
aOR (95% CI)	0.95 (0.82–1.08)	Ref	1.00 (0.88–1.12)
Group 4; BMI 23.0–< 25.0 (n = 5942)	n = 802	n = 2015	n = 3125
aOR (95% CI)	0.84 (0.58–1.22)	Ref	1.31 (1.04–1.62)
Group 5; BMI ≥ 25 (n = 5627)	n = 597	n = 1767	n = 3263
aOR (95% CI)	0.82 (0.57–1.19)	Ref	0.97 (0.78–1.21)
**UmA-pH < 7.1**			
Group 1; BMI < 18.5 (n = 9321)	n = 4664	n = 2871	n = 1786
aOR (95% CI)	1.30 (0.78–2.16)	Ref	1.49 (0.81–2.74)
Group 2; BMI 18.5–< 20.0 (n = 14,752)	n = 5261	n = 5294	n = 4197
aOR (95% CI)	1.04 (0.69–1.57)	Ref	1.46 (0.97–2.18)
Group 3; BMI 20.0–< 23.0 (n = 22,703)	n = 5807	n = 8099	n = 8797
aOR (95% CI)	0.83 (0.60–1.14)	Ref	0.96 (0.73–1.26)
Group 4; BMI 23.0–< 25.0 (n = 5942)	n = 802	n = 2015	n = 3125
aOR (95% CI)	1.42 (0.59–3.42)	Ref	2.08 (1.13–3.83)
Group 5; BMI ≥ 25 (n = 5627)	n = 597	n = 1767	n = 3263
aOR (95% CI)	0.29 (0.09–0.97)	Ref	0.80 (0.49–1.31)

Logistic regression models were used to calculate the adjusted odds ratios and 95% confidence intervals for UmA-pH < 7.20 and for UmA-pH < 7.10, with women with appropriate gestational weight gain as the reference. *BMI* body mass index, *UmA-pH* umbilical artery pH.

^a^Adjusted for maternal age, maternal education, annual household income, maternal smoking during pregnancy, parity, preterm birth and small-for-gestational age infants.

**Table 5:** Adjusted odds ratios^a^ and 95% confidence intervals for foetal acidosis based on gestational weight gain with caesarean section according to Japanese criteria^28^.

BMI category (kg/m^2^)	Gestational weight gain		
	Insufficient	Appropriate	Excessive
**UmA-pH < 7.2**			
Group 1; BMI < 18.5 (n = 1614)	n = 886	n = 445	n = 283
aOR (95% CI)	0.66 (0.40–1.10)	Ref	0.82 (0.43–1.56)
Group 2; BMI 18.5–< 20.0 (n = 2666)	n = 1049	n = 935	n = 682
aOR (95% CI)	1.00 (0.65–1.55)	Ref	1.05 (0.65–1.71)
Group 3; BMI 20.0–< 23.0 (n = 5132)	n = 1441	n = 1750	n = 1941
aOR (95% CI)	0.93 (0.67–1.29)	Ref	1.14 (0.85–1.52)
Group 4; BMI 23.0–< 25.0 (n = 1708)	n = 252	n = 607	n = 849
aOR (95% CI)	1.20 (0.64–2.23)	Ref	1.07 (0.67–1.71)
Group 5; BMI ≥ 25 (n = 2334)	n = 296	n = 721	n = 1317
aOR (95% CI)	1.04 (0.56–1.94)	Ref	1.19 (0.79–1.80)
**UmA-pH < 7.1**			
Group 1; BMI < 18.5 (n = 1614)	n = 886	n = 445	n = 283
aOR (95% CI)	0.51 (0.19–1.33)	Ref	1.43 (0.50–4.07)
Group 2; BMI 18.5–< 20.0 (n = 2666)	n = 1049	n = 935	n = 682
aOR (95% CI)	0.71 (0.35–1.45)	Ref	0.65 (0.28–1.49)
Group 3; BMI 20.0–< 23.0 (n = 5132)	n = 1441	n = 1750	n = 1941
aOR (95% CI)	1.29 (0.66 –2.51)	Ref	1.56 (0.86–2.84)
Group 4; BMI 23.0–< 25.0 (n = 1708)	n = 252	n = 607	n = 849
aOR (95% CI)	1.68 (0.56–5.01)	Ref	1.09 (0.44–2.72)
Group 5; BMI ≥ 25 (n = 2334)	n = 296	n = 721	n = 1317
aOR (95% CI)	1.63 (0.46–5.69)	Ref	1.84 (0.75–4.50)

Logistic regression models were used to calculate the adjusted odds ratios and 95% confidence intervals for UmA-pH < 7.20 and for UmA-pH < 7.10, with women with appropriate gestational weight gain as the reference. *BMI* body mass index, *UmA-pH* umbilical artery pH.

^a^Adjusted for maternal age, maternal education, annual household income, maternal smoking during pregnancy, parity, preterm birth and small-for-gestational age infants.

## Discussion

This study evaluated the risk of the actual GWG considering the pre-pregnancy BMI on foetal acidosis, while accounting for the mode of delivery. Excessive GWG in overweight women and women whose pre-pregnancy BMI was between 23.0 and < 25.0 kg/m^2^ increased the risk of foetal acidosis, especially in new-borns delivered vaginally, but not in those delivered by caesarean section. Conversely, excessive GWG did not have significant effects on foetal acidosis in women with the highest pre-pregnancy BMI in each classification (i.e. the IOM and Japanese criteria). Moreover, insufficient GWG in women with a pre-pregnancy BMI < 18.5 kg/m^2^ and in women with a pre-pregnancy BMI ≥ 25.0 kg/m^2^ (especially in those with vaginal deliveries) had lower risk of foetal acidosis than those with appropriate GWG.

The result of increased foetal acidosis in mothers with excessive GWG is consistent with previous studies^5^. Although previous studies have reported that maternal obesity increases the risk of foetal acidosis, these studies only included vaginal^17,19^ or caesarean^20^ deliveries, not both; no previous reports have shown the effects of GWG on foetal acidosis accounting for the mode of delivery. The increased prevalence of foetal acidosis with excessive GWG in women who underwent vaginal deliveries may have been caused by shoulder dystocia^21^ and otherwise traumatic labour due to foetal macrosomia, frequently seen in obese women^22^. Additionally, increased oxidative stress due to overweight status or obesity may affect the increased incidence of foetal acidosis^23^.

Conversely, excessive GWG did not affect foetal acidosis in every pre-pregnancy BMI group with caesarean delivery in the present study. Regarding caesarean section, high maternal BMI has been reported to increase operative difficulties, affect maternal haemodynamics, or influence pulmonary function intraoperatively, leading to decreased maternal oxygenation and placental perfusion^20^. Moreover, greater maternal BMI increases the risk of caesarean section due to foetal distress, which may increase the likelihood of foetal acidosis^24^. The discrepancy between the results of the present study and those of the study by Edwards et al.^20^ is uncertain. As obstetricians may prefer caesarean section to avoid adverse maternal and neonatal outcomes with expected difficulties in delivery, albeit without proper indications^25^, we speculate it is associated with the avoidance of difficulties in vaginal delivery in obese women, even though a higher number of participants had vaginal deliveries than caesarean deliveries in G4 and G5.

Furthermore, contrary to our expectation, excessive GWG in obese women and women whose BMI was ≥ 25.0 kg/m^2^ was not associated with an increased incidence of foetal acidosis. Although the reason underlying this result is also not clear, we speculate that in these patients, the initial bodyweight had a greater impact on foetal acidosis than did the GWG. Both appropriate and excessive GWG in these patients have an equivalent risk for foetal acidosis, which is supported by the statement that the lower the pre-pregnancy BMI, the stronger the association between increased GWG and birth weight in the 2009 IOM report^1^.

Consistent with a previous study that showed that bodyweight loss in obese pregnant women is associated with reduced perinatal risks^26^, this study showed an effect of insufficient GWG, including bodyweight loss, on reduced foetal acidosis. Insufficient GWG may solely decrease dystocia and operative difficulties in both vaginal deliveries and caesarean sections^20–22^, which may lead to decreased risk of foetal acidosis. Although insufficient GWG is desirable from the perspective of avoiding foetal acidosis, lean pregnant women and pregnant women with insufficient GWG may also exhibit adverse obstetric outcomes^27^. It is not advisable for physicians to suggest insufficient GWG for perinatal health care. However, it may be reasonable to suggest bodyweight loss for obese women to reduce perinatal risks.

The effect of GWG on foetal acidosis is greater in overweight women and women with a pre-pregnancy BMI of 23.0–< 25.0 kg/m^2^, rather than in those with obesity or BMI ≥ 25.0 kg/m^2^, respectively. Physicians should keep in mind the importance of appropriate bodyweight control during pregnancy to prevent foetal acidosis in these patients. It is also essential for care providers to note the impact of original pre-pregnancy bodyweight for patients with highest pre-pregnancy BMI in each classification, as well as the importance of proper bodyweight control before pregnancy. Moreover, physicians should be aware that the association between GWG and foetal acidosis is considerable with vaginal deliveries.

The present study used two different BMI criteria, the IOM guidelines^2–4^ and the Japanese described criteria^28^, because in Asian countries, including Japan, women have a lower pre-pregnancy BMI than do those in Western countries^29^. Even the World Health Organization (WHO) has proposed the use of a modified BMI threshold of ≥ 23 kg/m^2^ rather than ≥ 25.0 kg/m^2^ to define overweight conditions for Asians^30^. In Japan, obesity is defined as a pre-pregnancy BMI ≥ 25.0 kg/m^2^^28^, which differs from that of other countries^28,31^. The results showed same tendencies for the two different BMI classification schemes. Further studies are needed to determine the appropriate cut-off values for GWG to avoid foetal acidosis according to the specific pre-pregnancy BMI category and mode of delivery.

Additionally, because the JECS is a nationwide prospective birth cohort study, long-term neonatal and offspring outcomes can be analysed in the future. Although foetal acidosis is associated with neonatal mortality^10^ and foetal hypoxic-ischaemic brain injury^12^, long-term outcomes related to foetal acidosis increased by maternal GWG remain unclear. Further studies may clarify the effect of GWG on long-term neonatal and offspring outcomes in the JECS.

The present study’s main strength was the analysis of a large cohort of participants, in which the actual bodyweight change was calculated taking into consideration pre-pregnancy BMI categories using two different classification schemes^2–4,28^. In comparison, a recent study evaluating the increased risk of birth-asphyxia-related outcomes with GWG used changes in BMI and did not consider the pre-pregnancy BMI^5^.

The present study had several limitations. First, the mechanism of foetal acidosis, respiratory or metabolic, was not considered. The identification of metabolic acidosis is a key criterion for establishing a causal relationship between foetal perinatal asphyxia and neonatal encephalopathy and/or cerebral palsy^12,32^ because purely respiratory acidosis is not associated with neonatal adverse outcomes^32,33^. Here, foetal acidosis included foetal respiratory acidosis. Therefore, a careful interpretation of our findings and further studies of the mechanism underlying the association between GWG and foetal acidosis are needed. Second, detailed data about caesarean sections were lacking (i.e. data about the indication of caesarean section, elective caesarean section or emergent caesarean section, or vaginal delivery trial). These data may directly affect the foetal condition; thus, further studies are needed to clarify the effect of the background and pattern of caesarean section.

In conclusion, this study showed that excessive GWG in overweight women or women with a BMI ranging from 23.0 to < 25.0 kg/m^2^ was significantly associated with an increase in foetal acidosis, especially in vaginal deliveries, in a large Japanese cohort study. Conversely, excessive GWG did not have significant effects on foetal acidosis in women with the highest pre-pregnancy BMI in each classification (IOM and Japanese criteria). Further, an insufficient GWG in women with BMI < 18.5 kg/m^2^ and BMI ≥ 25.0 kg/m^2^, especially in those with vaginal deliveries, decreased the risk of foetal acidosis. Thus, proper counselling for appropriate gestational bodyweight control and the appropriate selection of the mode of delivery for women with excessive GWG are essential to avoid foetal acidosis.

## Methods

### Study design

We used data derived from the JECS, which is a nationwide prospective birth cohort study established in January 2011 to investigate the effects of environmental factors on children’s health^34,35^. Briefly, the JECS is funded directly by Japan’s Ministry of the Environment and involves collaboration between the Programme Office (National Institute for Environmental Studies), the Medical Support Centre (National Centre for Child Health and Development), and 15 regional centres (Hokkaido, Miyagi, Fukushima, Chiba, Kanagawa, Koshin, Toyama, Aichi, Kyoto, Osaka, Hyogo, Tottori, Kochi, Fukuoka, and South Kyushu / Okinawa)^35^. The eligibility criteria for the participants (expectant mothers) were as follows: (1) residing in the study areas at the time of recruitment and expected to continually reside in Japan for the foreseeable future; (2) an expected delivery date between August 1, 2011 and mid-2014; and (3) capable of participating in the study without difficulty (i.e. able to comprehend the Japanese language and complete the self-administered questionnaire).

Either or both of the following two recruitment protocols were applied: (1) recruitment at the time of the first prenatal examination at cooperating obstetric facilities; and (2) recruitment at local government offices issuing a pregnancy journal, called the Maternal and Child Health Handbook, which is given to all expectant mothers in Japan before they receive municipal services for pregnancy, delivery, and childcare. We contacted pregnant women via cooperating health care providers and/or local government offices issuing Maternal and Child Health Handbooks and registered those willing to participate. Self-administered questionnaires, which were completed by the women during the first trimester and second / third trimester, were used to collect information on demographic factors, medical and obstetric history, physical and mental health, lifestyle, occupation, environmental exposures at home and in the workplace, housing conditions, and socioeconomic status^35^.

The JECS protocol and the present study were reviewed and approved by the Ministry of the Environment Institutional Review Board on Epidemiological Studies and by the Ethics Committees of all participating institutions (Independent Ethics Committee (IEC) of the National Center for Child Health and Development, Hokkaido University, Institutional Review Board (IRB) of Sapporo Medical University, IEC of the Asahikawa Medical College, IEC of the Japanese Red Cross Hokkaido College of Nursing, IEC of Tohoku University, IEC of Fukushima Medical University, IRB of Chiba University, IEC of Yokohama City University, IEC of the University of Yamanashi, IEC of Shinshu University, The Ethics Committee of Toyama University, IRB of Nagoya City University, IEC of Kyoto University, The Doshisha University Research Ethics Review Committee Regarding Human Subject Research, IEC of Osaka University, IEC of Osaka Medical Center and Research Institute for Maternal and Child Health, IEC of Hyogo College of Medicine, IRB of Tottori University, The Research Ethics Committee of Kochi University, IRB of The University of Occupational and Environmental University -origination of this study, IEC of Kyushu University, IEC of Kumamoto University, IEC of the University of Miyazaki, IEC of the University of the Ryukyus). The JECS was conducted in accordance with the Helsinki Declaration and other national regulations and guidelines. All methods of this study were carried out in accordance with relevant guidelines and regulations. Written informed consent was obtained from all participating women. Informed consent was obtained from a parent or a legal guardian for participants below 20 years old.

### Data collection

The current analysis used the data set released in June 2016 (data set: jecs-ag-20160424). Specifically, we used three types of data: (1) M-T1, obtained from the first self-reported questionnaire that was collected during the first trimester, which included questions regarding the maternal medical background; (2) M-T2, obtained from the second self-reported questionnaire that was collected during the second or third trimester, which included partner lifestyle and socioeconomic status; and (3) Dr-0m, collected from medical records provided by each subject’s institution, which included obstetrical outcomes such as gestational age, birth weight, and UmA-pH.

Participants with singleton pregnancies after 22 weeks were included in the present study. Women with multiple pregnancies, abortion, still births, and missing information were excluded from the analysis. There were no significant differences in patient characteristics between those included and excluded (data not shown).

### Pre-pregnancy BMI, GWG, foetal acidosis, and confounding factors

Pre-pregnancy BMI was calculated according to the WHO standards (bodyweight [kg]/heigh^2^ [m^2^]). Information about pre-pregnancy weight and height was obtained by self-report in the first questionnaire. Participants were categorised using two previously established classifications. First, according to 2009 IOM guidelines, participants were categorised into four groups: GA: < 18.5 kg/m^2^, GB: 18.5–< 25.0 kg/m^2^, GC: 25.0–< 30.0 kg/m^2^ (overweight), and GD: ≥ 30.0 kg/m^2^ (obese). Second, according to the Japanese criteria described previously by Morisaki et al.^28^, participants were categorised into five groups: G1: < 18.5 kg/m^2^, G2: 18.5–< 20.0 kg/m^2^, G3: 20.0–< 23.0 kg/m^2^, G4: 23.0–< 25.0 kg/m^2^, and G5: ≥ 25.0 kg/m^2^. In Japan, obesity is defined as a pre-pregnancy BMI ≥ 25.0 kg/m^2^^28^, corresponding to G5 in our study. GWG was calculated as the bodyweight just before delivery minus the bodyweight before pregnancy (kg). Data on the bodyweight just before pregnancy were retrieved from medical record transcripts. Then, according to the pre-pregnancy BMI, GWG was categorised as ‘insufficient’, ‘appropriate’, or ‘excessive’ according to each classification (Supplementary Table S1)^2–4,28^.

Foetal acidosis was defined as an UmA-pH < 7.20 or < 7.10, according to a previous study that showed that a UmA-pH threshold of 7.20 was associated with an increased risk of adverse short-term outcomes^36^, and a second previous study, which showed that a UmA-pH threshold of 7.10 was associated with an increased risk of adverse neurological sequelae^11^.

The following items were considered as confounding factors: maternal age, maternal education, annual household income, maternal smoking during pregnancy, parity, PTB, SGA, and mode of delivery. Maternal age was categorised into three groups: < 20, 20–29, and ≥ 30 years based on a previous study, which showed that maternal age was associated with certain obstetric outcomes, such as PTB and SGA^37^. The educational status of the mother was categorised into four groups based on the years of education (junior high school: < 10, high school: 10–12, professional school or university: 13–16, and graduate school: ≥ 17 years). Annual household income was categorised into four levels (< 2,000,000; 2,000,000–5,999,999; 6,000,000–9,999,999; and 10,000,000 JPY). Maternal participants were requested to provide information about their smoking status by choosing one of the following: ‘continued smoking during pregnancy’, ‘never smoked’, ‘quit smoking before pregnancy’, and ‘quit smoking during early pregnancy’. Participants who chose ‘continued smoking during pregnancy’ comprised the smoking category, while the other participants comprised the non-smoking category. Parity obtained from the M-T1 data was categorised as nulliparous or multiparous. PTB was defined as delivery before 37 weeks. SGA was defined as a birth weight below − 1.5 standard deviations corrected for gestational age and sex according to the ‘New Japanese neonatal anthropometric charts for gestational age at birth’^38^. Mode of delivery was categorised as vaginal or caesarean delivery. These confounding factors were chosen based on clinical importance.

### Statistical analysis

Maternal characteristics were summarised according to the pre-pregnancy BMI category using two different classifications. One-way analysis of variance, Kruskal-Wallis test and the Chi-square test were used to compare continuous and categorical variables, respectively. After stratification by the pre-pregnancy BMI group, logistic regression models were used to calculate the aORs and 95% CIs for UmA-pH < 7.20 and < 7.10, with participants with appropriate GWG in each classification as the reference. In Model 1, maternal age, maternal education, annual household income, smoking, and parity were used to calculate the aOR to examine the effects of GWG on foetal acidosis. In Model 2, PTB and SGA, in addition to the adjusted factors in Model 1, were used to calculate the aOR for foetal acidosis. In Model 3, mode of delivery, in addition to the adjusted factors in Model 2, was used to calculate the aOR for foetal acidosis.

After this analysis, we stratified the participants in each BMI group according to the Japanese criteria based on the mode of delivery, and logistic regression models were used to calculate aORs and 95% CIs for foetal acidosis using the confounding factors of Model 2, as this model generated similar results to Model 1, but was considered to be of more importance.

SPSS version 26 (IBM Corp., Armonk, NY, USA) was used for the statistical analysis. A P value < 0.05 indicated statistical significance.

## Supplementary information

Supplementary Table S1.

## Data Availability

Data are unsuitable for public deposition due to ethical restrictions and the legal framework of Japan. It is prohibited by the Act on the Protection of Personal Information (Act No. 57 of 30 May 2003, amendment on 9 September 2015) to publicly deposit the data containing personal information. Ethical Guidelines for Epidemiological Research enforced by the Japan Ministry of Education, Culture, Sports, Science and Technology and the Ministry of Health, Labour and Welfare also restricts the open sharing of the epidemiologic data. All inquiries about access to data should be sent to: jecs-en@nies.go.jp. The person responsible for handling enquiries sent to this e-mail address is Dr. Shoji F. Nakayama, JECS Programme Office, National Institute for Environmental Studies.
