# Supplementary material for: Gestational weight gain and foetal acidosis in vaginal and caesarean deliveries: The Japan Environment and Children’s Study
Source: Sci Rep. 2020 Nov 23;10:20389. doi: 10.1038/s41598-020-77429-9 (PMC7683683; doi:10.1038/s41598-020-77429-9)
Supplement: Supplementary file 1 — Supplementary Table S1. [file 41598_2020_77429_MOESM1_ESM.pdf]

Gestational weight gain and foetal acidosis in vaginal and caesarean deliveries: The Japan Environment and Children's Study

Tsuyoshi Murata[1,2*]

Hyo Kyozuka[1,2]

Akiko Yamaguchi[1,2]

Toma Fukuda[1,2]

Shun Yasuda[1,2]

Akiko Sato[1]

Yuka Ogata[1]

Kosei Shinoki[1]

Mitsuaki Hosoya[1,3]

Seiji Yasumura[1,4]

Koichi Hashimoto[1,3]

Hidekazu Nishigori[1,5]

Keiya Fujimori[1,2]

and the Japan Environment and Children's Study (JECS) Group[21]

[1]Fukushima Regional Center for the Japan Environmental and Children's Study, 1

Hikarigaoka, Fukushima 960-1295, Japan

[2]Department of Obstetrics and Gynecology, Fukushima Medical University School of Medicine, 1 Hikarigaoka, Fukushima 960-1295, Japan

[3]Department of Pediatrics, Fukushima Medical University School of Medicine, 1 Hikarigaoka, Fukushima 960-1295, Japan

[4]Department of Public Health, Fukushima Medical University School of Medicine, 1 Hikarigaoka, Fukushima 960-1295, Japan

[5]Fukusshima Medical Center for Children and Women, Fukushima Medical University, 1 Hikarigaoka, Fukushima 960-1295, Japan

[21]A comprehensive list of consortium members appears at the end of the paper.

[*]**Corresponding author:** Tsuyoshi Murata

Department of Obstetrics and Gynecology, School of Medicine, Fukushima Medical University, 1 Hikarigaoka, Fukushima 960-1295, Japan

Tel.: +81-24-547-1290, Fax: +81-24-548-3878, E-mail: tuyoshim@fmu.ac.jp

Supplemental Table 1. Gestational weight gain categories stratified by pre-pregnancy BMI, in accordance with Morisaki et al. [28]

	gestational weight gain(kg)		
	insufficient	appropriate	excessive
pre-pregnancy BMI(kg/m^2)			
<18.5 (extremely lean)	<10.8	10.8-13.6	>13.6
18.5-19.9 (lean)	<9.5	9.5-12.4	>12.4
20.0-22.9 (normal)	<8.4	8.4-11.4	>11.4
23.0-24.9 (overweight)	<5.8	5.8-9.6	>9.6
25.0< (obese)	<1.7	1.7-6.9	>6.9

BMI; body mass index
